# Reevaluation of *FMR1* Hypermethylation Timing in Fragile X Syndrome

**DOI:** 10.3389/fnmol.2018.00031

**Published:** 2018-02-06

**Authors:** Hagar Mor-Shaked, Rachel Eiges

**Affiliations:** ^1^Stem Cell Research Laboratory, Medical Genetics Institute, Shaare Zedek Medical Center, Jerusalem, Israel; ^2^Hebrew University Medical School, Jerusalem, Israel

**Keywords:** Fragile X syndrome, *FMR1*, DNA methylation, CGG expansion, epigenetic gene silencing

## Abstract

Fragile X syndrome (FXS) is one of the most common heritable forms of cognitive impairment. It results from a fragile X mental retardation protein (FMRP) protein deficiency caused by a CGG repeat expansion in the 5′-UTR of the X-linked *FMR1* gene. Whereas in most individuals the number of CGGs is steady and ranges between 5 and 44 units, in patients it becomes extensively unstable and expands to a length exceeding 200 repeats (full mutation). Interestingly, this disease is exclusively transmitted by mothers who carry a premutation allele (55–200 CGG repeats). When the CGGs reach the FM range, they trigger the spread of abnormal DNA methylation, which coincides with a switch from active to repressive histone modifications. This results in epigenetic gene silencing of *FMR1* presumably by a multi-stage, developmentally regulated process. The timing of *FMR1* hypermethylation and transcription silencing is still hotly debated. There is evidence that hypermethylation varies considerably between and within the tissues of patients as well as during fetal development, thus supporting the view that *FMR1* silencing is a post-zygotic event that is developmentally structured. On the other hand, it may be established in the female germ line and transmitted to the fetus as an integral part of the mutation. This short review summarizes the data collected to date concerning the timing of *FMR1* epigenetic gene silencing and reassess the evidence in favor of the theory that gene inactivation takes place by a developmentally regulated process around the 10th week of gestation.

Fragile X syndrome (FXS; OMIM#300624) is one of the most common heritable forms of cognitive impairment (1 in 4000 male and 1 in 8000 female births), and is the leading known genetic cause of autism. It is inherited as an X-linked condition and results from a deficiency in the fragile X mental retardation protein (FMRP; McLennan et al., 2011). FMRP is an RNA-binding protein that is important for transport, stabilization and translation of mRNA into proteins that affect synaptic plasticity and connectivity in the central nervous system (Schaeffer et al., 2003; Santoro et al., 2011). In the absence of FMRP the dendritic spines are longer, thinner and less mature (Comery et al., 1997; Irwin et al., 2000).

Nearly all FXS patients lack FMRP due to an unusual loss-of-function mutation: a CGG tri-nucleotide repeat expansion in the 5′-UTR of the X-linked *FMR1* gene (Verkerk et al., 1991). As a function of the repeat tract size, four allele forms can be defined: normal (<45 CGG), intermediate (45–54 CGGs), premutation (55–200 CGG, PM) and full mutation (>200 CGG, FM) alleles (Rajaratnam et al., 2017). In the normal range, the copy number of CGGs is steady and the gene is fully functional. In rare cases, the number of CGGs increases slightly (intermediate). This has no effect on gene function. However, it increases the risk of further increase to the PM range in future generations. Individuals with PM do not manifest FXS but are prone to premature fragile X-associated ovarian failure (FXPOI) in females and fragile X-associated tremor/ataxia syndrome (FXTAS), particularly in males (Streuli et al., 2009; Gleicher and Barad, 2010). FXPOI is a primary ovarian defect characterized by absent menarche (primary amenorrhea) or premature depletion of ovarian follicles before the age of 40 years while FXTAS is a neurodegenerative condition that is characterized by adult-onset progressive intention tremor and gait ataxia. Both, FXPOI and FXTAS, have been attributed to RNA/protein gain-of-function mechanisms (Sellier et al., 2017).

When the CGGs expand to a length exceeding 200 repeats (FM) they become extensively unstable and result in FXS pathology. This occurs when the PM allele is transmitted by mothers but not fathers (Pembrey et al., 1985; McLennan et al., 2011). Once the CGGs reach the FM range they lead to *FMR1* epigenetic gene silencing through the induction of DNA methylation (Oberlé et al., 1991). Hypermethylation takes place through a specific pattern of acquisition. It spreads out from the 5′ flanking sequence (~650 bp upstream to the CGGs) to intron 1 of *FMR1*, spanning over the repeats and promoter region of the gene (Naumann et al., 2009). This occurs jointly with the loss of active histone modifications (H3K4me3) and the gain of repressive histone modifications (H3K9me2/3, H3K27me3), ultimately leading to a transcriptional block at the *FMR1* promoter (Coffee et al., 1999, 2002; Pietrobono et al., 2005; Tabolacci et al., 2005, 2008; Kumari and Usdin, 2010). Males with a FM are almost always severely affected by FXS, whereas females with a FM are generally less affected than males and manifest disease symptoms in only about 50% of the cases (Rajaratnam et al., 2017). This is because the *FMR1* gene is subject to X-inactivation in the somatic cells of females. A long-standing issue concerns the timing of *FMR1* epigenetic silencing in FXS. This is a fundamental question in the field of FXS research since the answer may provide new insights into the mechanism/s responsible for epigenetic gene silencing of *FMR1* in FXS.

Initially, when the gene for the disease had just been discovered, it was naturally assumed that hypermethylation was established and transmitted by the mother as an integral part of the FM. However, by monitoring for the repeat size and the methylation state of *FMR1* using methylation-sensitive Southern blot assays in fetal tissues and chorionic villus sampling (CVS) samples at 10–16 weeks of gestation, it became apparent that not all tissues are equally *FMR1*-hypermethylated during development. When the methylation status of the gene was examined, it was found to be heavily methylated in the majority of affected fetuses as early as 10 weeks of gestation, the time when embryo specification is already complete (Sutherland et al., 1991; Devys et al., 1992; Sutcliffe et al., 1992; Suzumori et al., 1993; Castellví-Bel et al., 1995; Willemsen et al., 2002). On the other hand, when the methylation status of *FMR1* was examined in the extra-embryonic tissues, the results were contradictory. While some reports found hypermethylation in CVS as early as 10 weeks (Devys et al., 1992; Suzumori et al., 1993), others showed that the *FMR1* is often hypomethylated and remains active even after 13 weeks of age (Sutherland et al., 1991; Sutcliffe et al., 1992; Castellví-Bel et al., 1995). In fact, this is why prenatal genetic diagnosis for FXS by CVS often leads to ambiguous results and much confusion that demands a follow-up via amniocentesis.

In a different study by Willemsen et al. (2002), the timing of *FMR1* gene silencing was determined by monitoring FMRP expression. This was achieved by immuno-histochemical analysis that provided an opportunity to monitor protein expression at the single-cell level while preserving tissue structure. The authors showed that FMRP gradually disappears in chorionic villi of XY affected fetuses between 10 weeks and 12.5 weeks of gestation. In CVS from 13 week old female fetuses with a FM, each villus was either completely positive or entirely negative for FMRP expression, implying that the proliferation of villi is a clonal process. Given that *FMR1* is an X-linked gene that is liable to X-inactivation, these findings suggested that the timing of *FMR1* gene silencing follows X-inactivation since no single villus contained a mixture of FMRP-expressing and non-expressing cells. Altogether, this led to the general impression that *FMR1* inactivation is an ongoing, developmentally regulated process initiated after embryo implantation, completed in the fetus by the end of first trimester, and frequently on hold in extra-embryonic tissues (Devys et al., 1992; Sutcliffe et al., 1992; Suzumori et al., 1993; Iida et al., 1994; Willemsen et al., 2002). In addition, it led researchers to conclude that CGG expansion is necessary, but certainly not sufficient for gene inactivation, and that additional differentiation-dependent factors are required to achieve epigenetic silencing.

The notion that the timing of repeat expansion and hypermethylation are different and that hypermethylation is achieved by a post-zygotic event, led to the assumption that FMs would be hypomethylated in fetal gametes. In fact, by probing for expansion size and *FMR1* methylation, Malter et al. (1997) provided evidence for the presence of unmethylated FM exclusively in intact ovaries of female fetuses (16 and 17 weeks gestation) by Southern blot analysis. Ruling out the possibility of the existence of PM alleles, they argued that the vast majority of oocytes in the ovaries harbor a FM in its unmethylated form. Given these data, it was presumed that repeat expansion may have already occurred in the female germ line, or very early during embryogenesis, prior to *de novo* methylation.

Although this study was limited to only two fetuses, it was consistent with the under-methylated state of the mutation in the extra-embryonic tissues of affected fetuses. In addition, it corresponded to known human embryo developmental milestones. During early embryogenesis, the precursor cells for the extra-embryonic tissues (trophectoderm, TE) and the primordial germ cells (PGCs) separate from the generally unmethylated epiblast just before genome wide *de novo* methylation takes place in the embryo proper at the time of implantation (Matsui and Mochizuki, 2014; Schroeder et al., 2015; Chatterjee et al., 2016). In mammals, DNA methylation patterns are initially established during gametogenesis. Almost all of these patterns are erased at the time of pre-implantation development, and then re-established by a second wave of methylation during early gastrulation, when the embryo initiates implantation (Kafri et al., 1992). During this second wave of methylation, CpG islands (such as the one in the 5′-UTR of *FMR1*) remain protected from methylation, allowing housekeeping genes to remain expressed in all cells of the embryo (Kafri et al., 1992). Therefore, it would be extremely beneficial if isolated oocytes with a FM (instead of intact ovaries) could be analyzed for the methylation status of the locus prior to fertilization. Such oocytes can be obtained during IVF procedures for women who are carriers of the fragile X FM/PM.

It should be noted that unlike female germ line cells, mature sperm cells with a FM have never been observed in adult males with FXS (Malter et al., 1997). This is because during fetal development FM alleles undergo contraction by a selection for this subpopulation of cells, ultimately resulting in the exclusive production of mature sperm cells with alleles in the PM range (Reyniers et al., 1993). This strongly suggests that gene silencing drives selection against FM alleles during spermatogenesis. If correct, this would imply that there is a major difference in the induction of *FMR1* hypermethylation between male and female germ lines. Conversely, it has been shown in *FMR1* knockout mice that FMRP is dispensable for spermatogenesis (Bakker et al., 1994). In an FXS family with a large deletion that hampers *FMR1* transcription, the deletion did not hinder male fertility (Meijer et al., 1994), once again supporting the hypothesis that FMRP-deficiency by *FMR1* hypermethylation does not impede spermatogenesis.

The idea that *FMR1* is inactivated fairly late during embryogenesis is not well supported by the high rate of methylation mosaicism observed in FXS human embryonic stem cell lines (hESCs; Avitzour et al., 2014). hESCs are derived from the inner cell mass (ICM) of blastocyst stage embryos (7-days post fertilization). As such, they reflect the cells in the embryo at the time of implantation. In recent years, over a dozen FXS hESC lines have been established from mutant IVF embryos that were obtained from high risk couples undergoing preimplantation genetic diagnosis procedures for FXS (reviewed in Mor-Shaked and Eiges, 2016). Initially, when the first male hESC line was established, it was found to express *FMR1* at normal levels and to be completely unmethylated at *FMR1* (Eiges et al., 2007). However, as more FXS hESC lines became available, it turned out that *FMR1* hypermethylation is not restricted to somatic cells in patients, but can also be acquired in the undifferentiated state (Avitzour et al., 2014; Colak et al., 2014). In fact, of the FXS hESC lines examined so far, the majority present some levels of methylation (Avitzour et al., 2014), although no line was observed with completely methylated (100%) or entirely transcriptionally inactive *FMR1*. Nevertheless, the finding that most of these cell lines are, at least in part, already methylated raises doubts as to the actual timing of epigenetic gene silencing in FXS. Perhaps *FMR1* hypermethylation is established before/at the time of embryo implantation; i.e., the developmental stage when the stem cell lines are established. In addition, since methylation levels remain largely unchanged over time in culture (more than 10 successive passages; Avitzour et al., 2014), unmethylated full expansions are most likely to arise from imperfect *de novo* methylation rather than from a failure to properly maintain aberrant methylation patterns. On the other hand, there is some evidence that when the size of the mutation drops below ~400 repeats but is still in the FM range (>200 repeats), methylation erodes (Zhou et al., 2016). This points to a threshold for the methylation of alleles bearing FM that may be higher than previously thought, and may change according to the type or differentiation state of the cell. To further corroborate these findings the threshold for methylation should be re-evaluated on a large sample of affected subjects. If correct, this will have major clinical implications for disease management and potential treatment. A higher threshold may also provide a plausible explanation for the lack of methylation acquisition in mice with more than 200 CGG repeats (Brouwer et al., 2007).

Interestingly, when examining female FXS hESC lines it was noted that like many other hESC lines, X-inactivation had already occurred in the majority of the cell lines, and was consistently skewed (Avitzour et al., 2014). Nevertheless, the nature of the skewing seemed to be unrelated to the activity of the gene. Whereas in some cell lines the maternal chromosome was inactivated, in others it was the paternal X. In addition, in certain cell lines where the normal X allele was inactivated, the repeat expansion was methylated whereas in others it was not. Therefore, and unlike in somatic cells of females with a FM (Salat et al., 2000), there appears to be no evidence for clonal selection of proliferating cells with FMRP expression in the undifferentiated state. This is in line with a previous report that selection for the unmethylated allele through skewed X-inactivation in FM carrier females is the most strongest between birth and puberty (Godler et al., 2013). The inverse correlation between age and the proportion of active X chromosomes harboring the FM in these females (Rousseau et al., 1991) further supports the undifferentiated state lack of clonal selection hypothesis.

Given that X-inactivation has already been induced in most female cell lines, hESCs may more closely resemble epiblast (primed ESCs) than the ICM cells (naïve ESCs) in the embryo. This is because X-inactivation does not initiate in the embryo until implantation, when the ICM converts into the epiblast. If so, these undifferentiated immortalized cell lines should reflect a less primitive ground state of pluripotency and exhibit higher methylation levels than originally assumed. In fact, in an earlier study researchers reported they had been able to re-activate the *FMR1* gene in FXS patients’ cells with a hypermethylated full expansion by creating induced pluripotent stem cells (iPSCs) through transcription factor reprogramming (Gafni et al., 2013). By growing the cells in naïve supportive conditions they observed promoter CpG demethylation and upregulation of *FMR1* mRNA levels. However, under similar conditions other researchers failed to reverse/prevent *FMR1* hypermethylation in FXS iPSCs clones, respectively (de Esch et al., 2014). Careful examination of the parental cells in the earlier study indicated mosaicism for expansion size. Hence, it would be crucial to re-examine the size of the expansion following reprogramming before drawing any conclusions. In addition, it would be equally important to explore the methylation status of the gene in FXS hESCs lines which were derived under naïve conditions from the very beginning. If *FMR1* is found to be consistently hypomethylated, the switch from naïve to primed hESCs, and vice versa, could provide a powerful tool to both trace and intervene in the process of *FMR1* epigenetic gene silencing by turning on and off the gene in a reversible fashion.

In any case, since the majority of FXS patients present heavily methylated expansions in their soma, it is expected that a second wave of *de novo* methylation will take place by cell differentiation. However, reports on the effect of *in vitro* differentiation into neurons on the methylation status of the mutation are contradictory. One study reported epigenetic silencing achieved by a switch from active (H3K4me2) to repressive (H3K9me2) histone modifications by day 45 of neuronal differentiation (Colak et al., 2014), whereas in others neuronal differentiation of up to 90 days failed to trigger epigenetic silencing (Brykczynska et al., 2016; Zhou et al., 2016). In fact, it is becoming increasingly clear that the conversion of an active unmethylated FM allele into a silenced one appears to be conditioned on an increase in the length of the CGG tract rather than on the differentiation status of the cell. This may imply that unmethylated FM are not subject to epigenetic silencing in a differentiation-dependent fashion as is commonly thought. Instead, there is some indication that *FMR1* hypermethylation may be gradually achieved through selection against unmethylated FM rather than by an active mechanism following cell specification (Zhou et al., 2016). In fact, in the earlier study on the induction of *FMR1* epigenetic silencing by neural differentiation (Colak et al., 2014), careful examination of the expansion by Southern blot analysis indicated certain levels of methylation on the FM alleles to begin with. Since unmethylated FM coincides with the formation of RNA foci in the nuclei of *in vitro* differentiated cells (Brykczynska et al., 2016), it would be useful to show that this subpopulation of cells is eventually eliminated in the embryo by an RNA gain-of-function mechanism. Indeed, there is increasing evidence that lengthy mRNAs are toxic to the cells and may be the leading cause of FXPOI (Elizur et al., 2014; Man et al., 2017) and FXTAS (Galloway and Nelson, 2009; Li and Jin, 2012) in PM carriers. It is expected that longer transcripts with greater number of repeats should have a more deleterious effect leading to cell death at a much earlier stage during development.

Importantly, contrary to original assumptions, hypermethylation of *FMR1* in FXS male patients is recurrently not complete. The degree of methylation can differ between or within different tissues in the same individual, giving rise to inter- or intra-tissue mosaicism (Dahl and Guldberg, 2007; Chen et al., 2011). These unmethylated alleles are transcriptionally active, and in many cases over-express *FMR1* (Tassone et al., 2001). Although partial methylation patterns in somatic cells of patients have been reported (Stöger et al., 1997; Dahl et al., 2007; Chen et al., 2011), the rate of such mosaicisms has not been extensively studied, and it is commonly believed to be infrequent. However, most of the methylation analyses performed on patients’ cells, as well as the earlier studies with human fetal tissues and gametes were done using methylation-sensitive Southern blot assays. The limited sensitivity of Southern blot assays to accurately determine methylation levels near and at the CGG repeats is most likely the cause of the underestimated frequency of methylation mosaics in FXS patients and fetuses. Based on the analysis of 20 FXS patients using a PCR-based method (methylation-specific melting curve analysis), Dahl and Guldberg (2007) reported that 20% of the patients were actually methylation mosaics. Published and unpublished data suggest that this is actually a much more widespread event. The high rates of methylation mosaics are in line with the fact that the majority of the patients (60%) express significant levels of *FMR1* mRNA although none of them carry any PM alleles (Tassone et al., 2001). This is consistent with the heterogeneous levels of aberrant methylation observed in FXS hESC lines (Avitzour et al., 2014), and corresponds to the inability of FM to actively acquire hyper-methylation following cell differentiation *in vitro* (Brykczynska et al., 2016; Zhou et al., 2016).

To account for the data related to: (1) extensive *FMR1* methylation in fetal tissues by the end of the first trimester; (2) the high rates of methylation mosaics among male patients; and (3) the wide variability in the epigenetic status of the expanded gene between and within the currently available FXS XY hESC lines, we proposed a temporal model of *FMR1* hypermethylation (Figure 1) which suggests that abnormal methylation is first acquired stochastically on full expansions during a restricted point in time before or during embryo implantation. Once established, it is clonally maintained. Expansions which escape abnormal methylation during this limited time frame remain unmethylated, and most likely are eliminated by a toxic RNA gain-of-function mechanism or are actively methylated as an outcome of cell differentiation. It would be extremely useful to explore whether FXS pre-implantation embryos are already *FMR1* methylated. If so, it would be just as important to determine whether methylation is uniformly induced in all cells of the embryo before the stage of stem cell line derivation. In addition, it would be key to extend the analysis to a greater number of oocytes by looking at cumulus-stripped mature eggs using more delicate methylation-sensitive assays. This would further strengthen the widely held assumption that methylation is beyond doubt a post-zygotic event. Alternatively, methylation may be established in the female germ line and transmitted to the fetus as an integral part of the mutation, but not properly maintained in the early embryo while the overall levels of the DNMT1 (maintenance methylase) are generally low.

**Figure 1:**
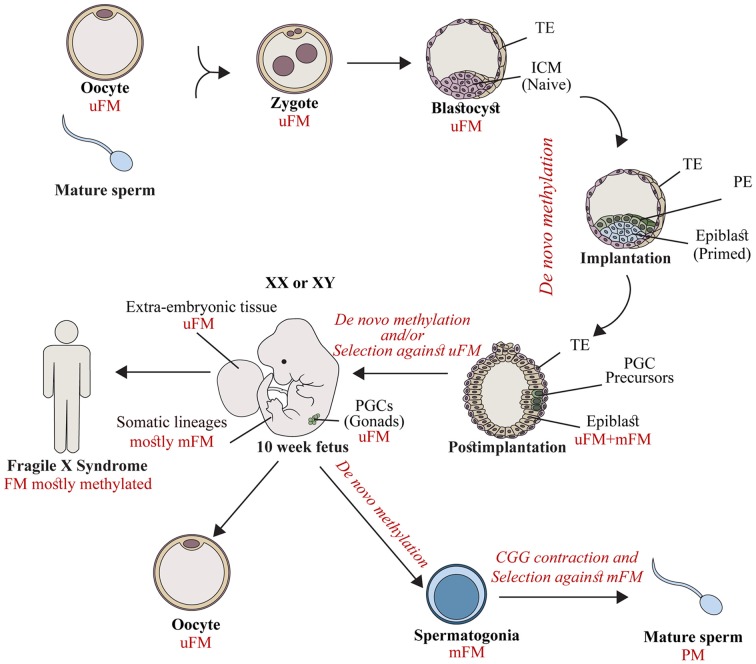
The timing of *FMR1* gene silencing during development. Fragile X syndrome (FXS) results from full mutations (FMs) which are exclusively transmitted by mothers in their unmethylated form (uFM). Hypermethylation is first established stochastically before/at the time of embryo implantation. This occurs in the inner cell mass (ICM, naïve cells) or the epiblast (primed cells) of affected fetuses, during the developmental stages when embryonic stem cell lines are established. FM alleles remain unmethylated in primordial germ cells (PGCs) precursors, and extra-embryonic tissues. Later during development, a second wave of *de novo* methylation takes place (postimplantation to 10 week-old fetuses). Hypermethylation coincides with a selection against cells with an uFM, and results in *FMR1* gene silencing in the majority of fetal tissues (10–13 weeks of age) and in the soma of FXS affected individuals. When the PGCs initiate differentiation they experience a third wave of *de novo* methylation in the male germ line. FM alleles become methylated (spermatogonia) and, as a result are eliminated. Otherwise they contract, resulting in the exclusive production of mature sperm cells with alleles in the premutation (PM) range (mature sperm). This is different from the female germ line, where FMs remain unmethylated, awaiting the time of fertilization. TE, Trophectoderm; PE, primitive endoderm.

There are a number of other avenues for further exploration in the area of epigenetic gene silencing of *FMR1*. It would be worthwhile exploring whether hypermethylation is induced by the conversion of mutation-bearing cells from the naïve to the primed pluripotent state. It would also be interesting to explore whether a second wave of *de novo* methylation occurs as a result of cell differentiation, or is a consequence of negative selection of cells bearing unmethylated FM over methylated FM alleles. Finally, it would be prudent to resolve the question of whether methylation plays a role in restricting repeat instability, and if so how the timing of *de novo* methylation impacts expansion size in the fetus as well as gamete precursors.

## Author Contributions

HM-S and RE contributed to the conception, design and writing of this manuscript.

## Conflict of Interest Statement

The authors declare that the research was conducted in the absence of any commercial or financial relationships that could be construed as a potential conflict of interest.
